# Papyrus: a large-scale curated dataset aimed at bioactivity predictions

**DOI:** 10.1186/s13321-022-00672-x

**Published:** 2023-01-06

**Authors:** O. J. M. Béquignon, B. J. Bongers, W. Jespers, A. P. IJzerman, B. van der Water, G. J. P. van Westen

**Affiliations:** Division of Drug Discovery and Safety, Leiden Academic Centre for Drug Research, Leiden University, Leiden, The Netherlands

**Keywords:** Machine learning, Cheminformatics, Bioactivity, Curated dataset, Papyrus, Standardisation, Normalisation

## Abstract

With the ongoing rapid growth of publicly available ligand–protein bioactivity data, there is a trove of valuable data that can be used to train a plethora of machine-learning algorithms. However, not all data is equal in terms of size and quality and a significant portion of researchers’ time is needed to adapt the data to their needs. On top of that, finding the right data for a research question can often be a challenge on its own. To meet these challenges, we have constructed the Papyrus dataset. Papyrus is comprised of around 60 million data points. This dataset contains multiple large publicly available datasets such as ChEMBL and ExCAPE-DB combined with several smaller datasets containing high-quality data. The aggregated data has been standardised and normalised in a manner that is suitable for machine learning. We show how data can be filtered in a variety of ways and also perform some examples of quantitative structure–activity relationship analyses and proteochemometric modelling. Our ambition is that this pruned data collection constitutes a benchmark set that can be used for constructing predictive models, while also providing an accessible data source for research.

**Graphical Abstract:**

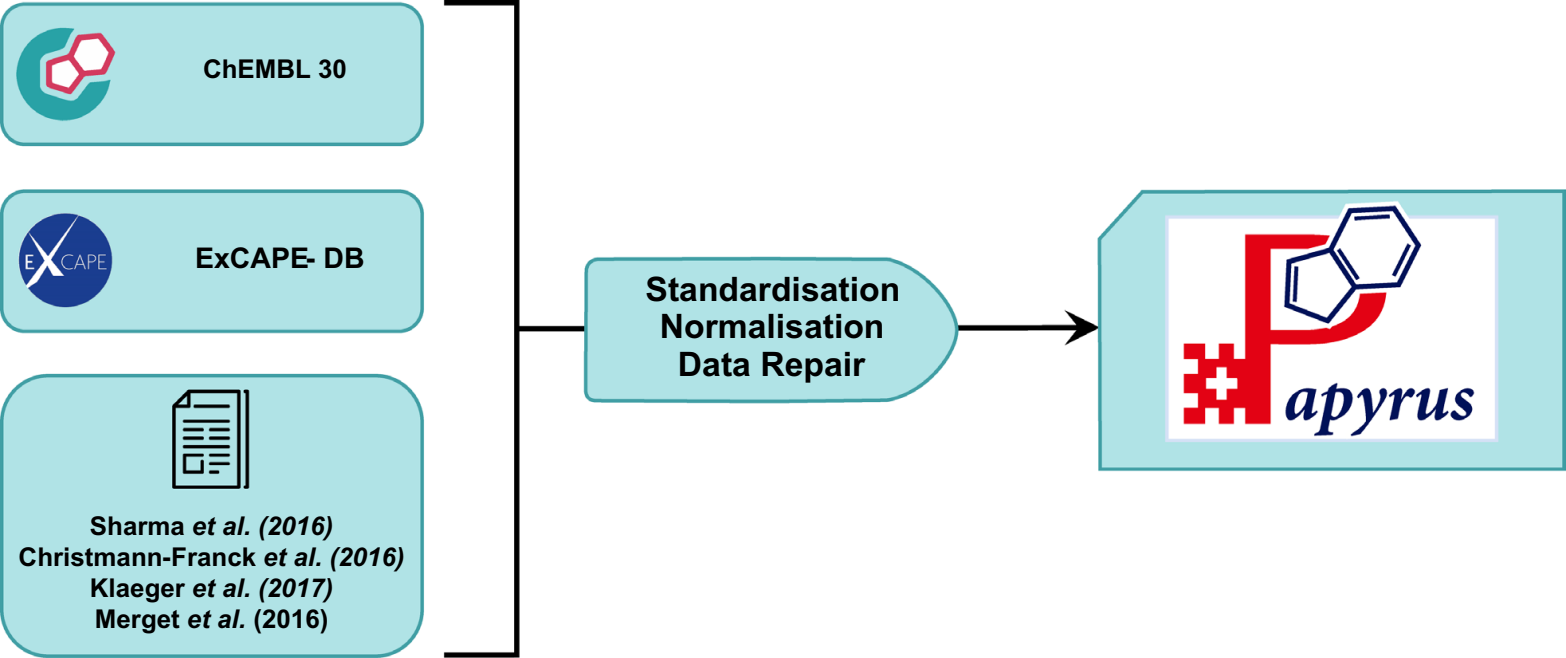

**Supplementary Information:**

The online version contains supplementary material available at 10.1186/s13321-022-00672-x.

## Introduction

Academic computational drug discovery has been expanding rapidly along with the growth of publicly available data [1, 2]. One of the areas with accelerated developments is the prediction of bioactivity, specifically the prediction of ligand–protein affinity. Databases such as ChEMBL [3], PubChem BioAssays [4], PDBBind [5, 6] or BindingDB [7] provide a wealth of information on ligands, proteins, and their interaction. Others focus on toxicological endpoints but contain seldom protein–ligand interaction data such as Tox21 [8] or ToxCast [9, 10]. However, public data have a diverse quality range and are subject to experimental error [11, 12]. In contrast to large datasets like ChEMBL, there are also smaller, more focused datasets available. These typically focus on a single protein family and are obtained from a single paper such as the Klaeger clinical kinase drugs dataset [13]. Such collections contain a trove of high-quality data, but are limited in their scope and typically lack metadata and inclusion of generally applicable identifiers. Some other works have also focused on the removal of unselective hits from data of the PubChem BioAssays but resulted in a selection limited to 18 protein assays only [14] and were shown to bias models towards memorization rather than generalization [15]. Building on this last observation the LIT-PCBA was designed to filter out assay artefacts but consists of bioactivity data towards only 15 targets [16]. Recently, a dataset called MolData, addressing most of the shortcomings aforementioned and containing 103,440,515 bioactivity data points was published [17]. Nonetheless, the bioactivities were binarized hence preventing their use for regression tasks.

In previous work, the performance of established bioactivity prediction methods was compared to that of deep neural networks [18]. A public dataset was devised for this work, relying on ChEMBL (version 20) from which a high-quality subset was extracted and made available [19]. Though initially planned, other smaller-scale datasets could not be included due to the amount of work needed to prepare the ChEMBL dataset. In addition, the selection of high-quality data reduced the size to 2.5% of the total ChEMBL data.

The current research aims to address these issues and to produce a standardised dataset. This dataset, named Papyrus [20] (in reference to Leiden Papyrus X [21]), is created with ease-of-use and filtering in mind such that it can be used ‘out-of-the-box’. Aside from ChEMBL version 30 (ChEMBL30), data from the ExCAPE-DB [22] database were added, along with the focused Sharma et al*.*’s [23], Christmann-Franck et al.’s [24], Klaeger et al*.*’s [13] and Merget et al*.*’s [25] datasets. Additionally, a data quality annotation was devised for each compound-target pair, characterizing the quality of the machine learning models obtained if trained from them. Moreover, correspondences to the Protein Data Bank’s [26] three-dimensional structural data were identified to allow for easier bridging of ligand- and structure-based modelling. Furthermore, high-quality protein–ligand interactions were modelled for adenosine receptors (ARs), C–C chemokine receptors (CCRs), kinases, monoamine receptors (MRs) and solute carrier 6 transport family (SLC6). Baseline performances of machine learning quantitative structure–activity relationship (QSAR), proteochemometric (PCM) and single-task deep neural networks (DNN) models are reported to demonstrate the quality of the proposed dataset, both using a random and temporal data split. Finally, a filtered version called Papyrus++ was devised, considering only data of reproducible assays.

## Material and methods

### Construction of Papyrus

The Papyrus dataset was obtained by collecting and processing ChEMBL’s (version 30) 19,286,751 activity data points measured on 2,157,379 compounds and 14,855 targets, ExCAPE-DB’s 70,850,163 activity data points of 998,131 compounds measured on 1667 targets, Sharma et al.’s dataset [23] of 258,060 activity data points of 76,017 compounds measured on 8 targets, Christmann-Franck et al*.*’s dataset [24] of 344,788 activity data points of 2065 compounds measured on 448 targets, Klaeger et al*.*’s dataset [13] of 5916 activity data points of 243 compounds measured on 520 targets and Merget et al*.*’s dataset[25] of 260,757 activity data points of 47,774 compounds measured on 341 targets. The data was standardized and filtered after which 59,775,087 activity values associated with 1,270,570 unique two-dimensional compound structures and 6926 proteins were obtained.

Complete preparation steps taken to create the Papyrus dataset are available in Additional file 4 and parameters in Additional file 2: Tables S1–S13. Briefly, only data associated with Ki, K_D_, IC_50_, EC_50_ and their logarithm transforms were considered if expressed in molar concentrations, molecules structures were standardized using ChEMBL structure pipeline [27] as well as a combination of OpenBabel [28, 29], tautomer canonicalization and Dimorphite-DL [30]. Proteins were mapped to UniProt [31] identifiers, sequences, and ChEMBL’s tiered protein classification.

Throughout the filtering and standardization process, the data were prepared considering three levels of quality for machine learning: the data regression models can be developed from are labelled high-quality while those classifiers can model are labelled low-quality. Medium quality is available for regression models and is associated with bioactivity data points associated with lower quality of the associated bioassays.

### Construction of Papyrus++

In addition to the Papyrus dataset, a high-quality version was devised, termed Papyrus++. It was obtained by keeping data points associated with Ki and K_D_ measurements intact and by filtering IC_50_ and EC_50_ data as follows. For IC_50_ and EC_50_ values separately measurements of compound-target pairs across different assays were filtered out if their respective absolute distance to the median was greater than 0.5 log units, then considered non-concordant. If a compound-target pair was associated with only one IC_50_/EC_50_ data point, it was included only if its assay was deemed reproducible (i.e. was concordant to other assays based on different compound-target pairs 75% of the time).

### Use of Papyrus

The subsets from the application examples were extracted from a prior version of the Papyrus dataset that included ChEMBL version 29 instead of 30.

#### Data subset extraction

The first subset that was extracted from Papyrus consists of adenosine receptors ARs. Using the Papyrus Python scripts, data of high quality with protein classification level 5 being “Adenosine receptor” were extracted. This subset consisted of 15,941 activity points, 24 protein targets, and 7967 compound structures. Human kinases data were similarly extracted using protein classification level 2 of “Kinase”, with 264,350 activity points, 476 protein targets and 91,556 compound structures. A total of 13,013 activity values from 33 protein targets and 7254 compound structures were retrieved for the SLC6 transporters by setting the protein classification level 4 filter to “SLC06 neurotransmitter transporter family”. Human CCRs were filtered with protein classification level 5 set to “CC chemokine receptor” and resulted in a subset of 4778 activities associated with 11 protein targets and 4681 compounds. Finally, the subset of human MRs was filtered using a protein classification level 4 set to “Monoamine receptor” and consisted of 41,482 activity values, 37 protein targets and 22,460 compound structures.

#### Matching the Protein Data Bank

To extend binding affinity data in the Papyrus dataset with experimentally determined 3D structures of protein–ligand complexes a script was devised that matches the Protein Data Bank [26] ligands to Papyrus data via their international chemical identifiers (InChI) [32] and proteins UniProt accession codes. Data from the Protein Data Bank were retrieved using the REST API identifier mapping service. Mutations introduced to the experimentally determined structure were not taken into consideration, thus the structures were mapped to the affinity data for the wild-type protein. If multiple structures of the same protein–ligand complex were found, all were retrieved.

#### Data visualisation

Unique molecules of Papyrus were collected based on the uniqueness of their connectivity. Each molecule was encoded using MinHash fingerprint (MHFP6) [33] and then visualised using TMAP [34]. Molecules were labelled using the initial dataset they originated from [33].

#### Diversity analysis of molecular structures

Molecular diversity was determined using sphere exclusion diversity [35]. Extended connectivity Morgan fingerprints with radius 3 (ECFP6) and 1024 bits were calculated with the RDKit [36] for each molecule. The leader algorithm variation of the sphere exclusion algorithm [37] implemented by Roger Sayle in the RDKit [38] was then used with a sphere radius set to 0.65 Tanimoto distance. To normalise for the size of the datasets, subsets of 228 compounds—the number of standardised compounds in the Klaeger dataset—were randomly picked 10,000 times from each dataset. The sphere exclusion diversity was defined as the fraction of diverse compounds selected by the leader algorithm. This process was then repeated disregarding the Klaeger dataset and using subsets of 1500 compounds randomly picked 10,000 times from each dataset. For comparison, we included subsets of enumerated virtual libraries of stable molecules up to 17 heavy atoms (50 million molecules) [39] and 13 heavy atoms (1 million molecules) [40], and a synthetically accessible diversity-orientated virtual library (Enamine diverse) of 50,240 molecules.

#### Bioactivity modelling: quantitative structure–activity relationships

Each protein target in the subset was modelled independently using the Papyrus Python scripts to obtain a machine learning-based QSAR model. Several targets were disregarded for modelling when less than 30 active and inactive compounds, based on the activity threshold of 6.5, were present or when associated with activity values spanning less than 2 log units. Then for each target, a random and a temporal split between training and hold-out test sets were performed. For the temporal split, data points associated with the year 2013 and above constituted the test set. If no activity data was available either before, on or after the year 2013, then the target was disregarded. The 777 Mold2 molecular descriptors [41], 512-dimensional continuous data-driven descriptors (CDDD) [42], 1613 Mordred two-dimensional molecular descriptors [43], and the RDKit [36] ECFP6 with 2048 bits were calculated for each molecule. All descriptors but ECFP6 bits were centred and scaled to unit variance. Extreme Gradient Boosting [44] (XGBoost version 1.4.2) regressors and classifiers were trained on randomly split training sets using random seed 1234 and default parameters. Regressors were trained to predict mean pChEMBL values using fivefold cross-validation, while classifiers were trained to predict a binary label of activity class with a threshold set at 6.5 log units using fivefold stratified cross-validation.

#### Bioactivity modelling: proteochemometrics

No subsequent filtering of the subsets was carried out since PCM handles multiple targets all at once. A temporal split on the year 2013 was employed to split the training and test set. Proteins were described using the concatenation of UniRep [45] 64, 256 and 1900 average hidden states, final cell states, and final hidden states, resulting in 6660-dimensional protein descriptors. XGBoost classifiers and regressors were trained using the same protocol as for QSAR models.

#### Bioactivity modelling: Deep Neural Nets

Single-task PCM DNN models were created using PyTorch [46] 1.10.0 with CUDA toolkit version 11.3.1. Models consisted of three hidden fully connected layers with 8000, 4000 and 2000 neurons respectively. The binary cross-entropy was used as a loss function for classifiers along with a sigmoid activation function while the mean-square error was used for regressors along with a rectified linear unit activation function. The Adam optimizer [47] was used to optimize the loss with a learning rate of 10^–3^. Proteins were represented by a concatenation of the final cell state, final hidden state and average of hidden states of UniRep representations each with 256 dimensions. The training process lasted for 1000 epochs with early stopping after 300 epochs, 25% of hidden neurons were randomly dropped out between each layer and a batch size of 1024 was used. For the kinase and monoamine receptor subsets, the dimensions of the protein descriptors were reduced and consisted of the concatenation of the UniRep final cell state, final hidden state and average of hidden states each with 64 dimensions, early stopping was set to 20 epochs and batch size decreased to 64.

## Results and discussion

A new dataset of bioactivities, called Papyrus, resulting from the aggregation and extensive standardisation of data from six sources, was created. Unless mentioned otherwise, only the extensively standardised Papyrus set without stereochemistry is considered in this section.

### Papyrus dataset statistics

The Papyrus dataset consists of 59,775,087 compound-protein pairs, each associated with at least either one activity value or activity class. Additionally, this represents the data of 1,270,570 unique two-dimensional compound structures and 6926 proteins across 499 different organisms. In terms of data quality, 1,238,835 data points are of high quality, i.e., representing exact bioactivity values measured and associated with a single protein or complex subunit. 335,661 data points are of medium quality, i.e., exact bioactivity values associated with either potentially multiple proteins or a homologous single protein. 58,200,591 data points are of low quality, i.e., exact bioactivity values associated with either multiple homologous proteins or homologous complex subunits, censored bioactivity values and binary activity classes. When considering data points across all quality types, 2,585,248 are associated with exact bioactivity values, 354,981 with censored data and 56,823,552 with binary activity classes. The repartition of data quality across the ten organisms with the most data (Table 1) indicates a clear bias towards humans, with 55,595,516 data points or more than 93% of the data related to it, but also emphasizes the interest towards rodent targets with 2,513,821 data points or more than 4% of the data associated with mouse and 1,244,385 data points or 2% with rats.

**Table 1:** Activity data of organisms in Papyrus with the most data points

Species	Quality			Total	% of total
	High	Medium	Low		
*Homo sapiens* (Human)	987,436	246,401	54,364,908	55,598,745	93.01
*Mus musculus* (Mouse)	42,078	6682	2,465,157	2,513,917	4.21
*Rattus norvegicus* (Rat)	60,475	32,061	1,151,955	1,244,491	2.08
*Escherichia coli* (strain K12)	539	11,298	60,030	71,867	0.12
*Equus caballus* (Horse)	18,330	32	27,988	46,350	0.08
Influenza A virus (A/WSN/1933(H1N1))	23,813	–	9143	32,956	0.06
*Trypanosoma cruzi*	5935	30	23,927	29,892	0.05
*Schistosoma mansoni* (Blood fluke)	13,916	–	14,473	28,389	0.05
*Bacillus subtilis*	12,106	–	11,693	23,799	0.04
*Bos taurus* (Bovine)	5944	5105	8918	19,967	0.03

When it comes to the activity types the Papyrus dataset is derived from (Table 2), most of the data are either associated with untraceable data types, such as for binary data, or with types derived from others—for instance, the KIBA scores were derived from IC_50_, K_i_ and K_D_ data [12] present in the Merget source dataset.

**Table 2:** Number of original data points in Papyrus for each activity type

Activity type	Original data points
K_i_	509,022
K_D_	119,455
IC_50_	1,082,403
EC_50_	142,251
Other	58,314,761

**Fig. 1:**
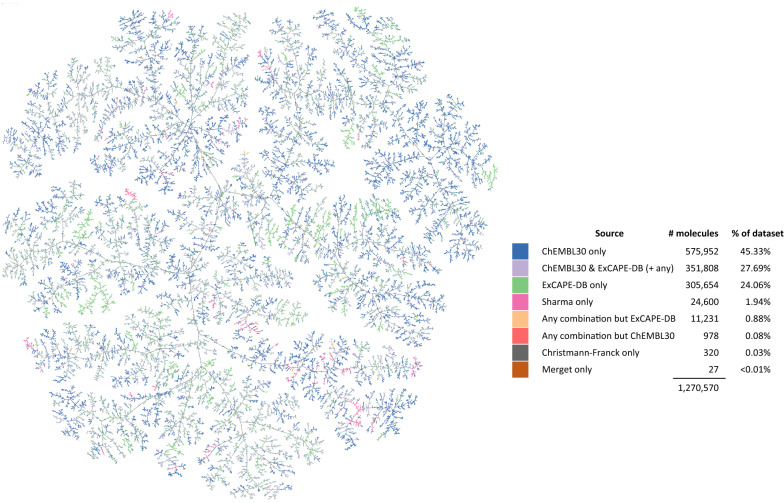
TreeMap of the Papyrus chemical space. Though some local branches are enriched in compounds of a specific subset, no clear global region of the chemical space is dominated by a specific dataset

**Fig. 2:**
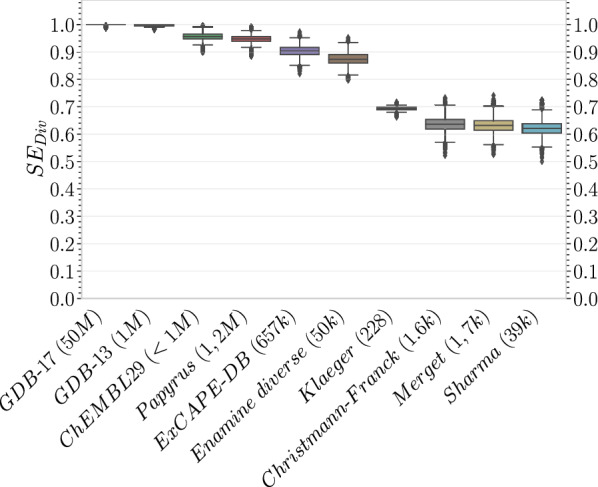
Sphere exclusion diversity (SE_Div_) of randomly sampled subsets of 228 molecules of the Papyrus dataset, its source subsets and reference virtual libraries GDB-17, GDB-13 and Enamine synthetically accessible diversity set

Visualising the compound space of the Papyrus dataset (Fig. 1) revealed that, while the dominant space was led by ChEMBL and ExCAPE-DB, there were no defined regions mostly associated with one source or the other, suggesting that their respective data complemented each other. In order to estimate the diversity of molecules in Papyrus, the sphere exclusion diversity (SE_Div_) recently proposed by Thomas et al*.*, which aligns better with chemical intuition than the average Tanimoto similarity, was employed (Fig. 2). This diversity measure corresponds to the fraction of cluster centres picked by the sphere exclusion clustering algorithm out of the considered set of molecules. The authors interpret this as the minimum fraction of the dataset required to explain the chemical diversity in the context of bioactivity [35]. Using a threshold of 0.65 (i.e., Tanimoto similarity of 0.35 or above) broadly correlates to an 80–85% probability of belonging to the same bioactivity class. SE_Div_ of the Papyrus dataset was compared to that of the subsets it is composed of along with reference virtual libraries GDB-13 and GDB-17 and the Enamine synthetically accessible diversity set, using random subsamples of 228 molecules. The SE_Div_ of the GDB-17 and GDB-13 rank first with values close to 1.0, as expected from databases of such sizes. Interestingly, the SE_Div_ of the Papyrus dataset lies between that of ChEMBL30 and ExCAPE-DB despite being composed of both, with average SE_Div_ values of 0.95, 0.96 and 0.90 respectively. This is an indication that certain chemical series which seem more ‘*popular*’ than others are shared among the datasets composing Papyrus. Additionally, the Klaeger dataset, with its 228 compounds, was identified as being more diverse than the Christmann-Franck, Merget and Sharma datasets which were larger in size (7–170 times bigger), with average SE_Div_ values of 0.69, 0.64, 0.63 and 0.62 respectively. Conducting the same analysis disregarding the Klaeger dataset and using random subsamples of 1500 molecules led to the same ranking of datasets’ diversities (Additional file 1: Fig. S1).

**Fig. 3:**
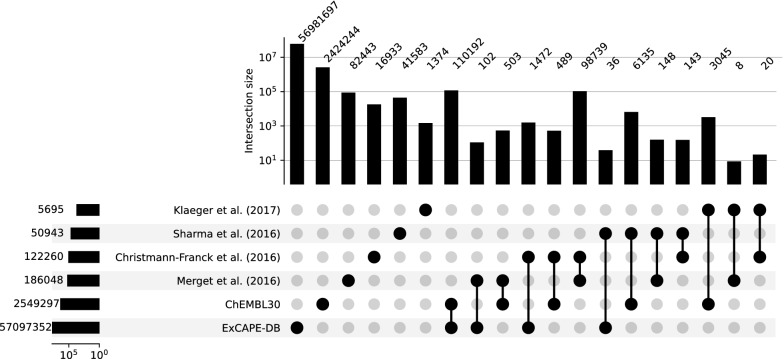
Activity overlaps between the aggregated datasets. Shown on the left side is the number of protein–ligand interaction activity points in each dataset. The numbers on the top refer to the number of these activity data points found in that particular dataset or overlap of datasets. Overlaps of more than two datasets can be found in Figure S2 along with protein target and chemical space overlap

To further illustrate the complementarity of the datasets composing Papyrus, overlaps of the activities in these datasets were determined (Fig. 3). Although ExCAPE-DB provides the most amount of unique activity data, all sets bring non-overlapping data to the full Papyrus set. The most notable overlaps, when omitting that between ExCAPE-DB and ChEMBL30 of 110,192 activity values, are between Merget and Christmann-Franck (98,739 overlapping points) representing 53.1% and 80.8% of each dataset respectively, as they provide high-quality data on the same targets, between Sharma and ChEMBL30 (6135 overlapping points) representing 12.0% and 0.2% of each dataset respectively, and between Klaeger and ChEMBL30 (3045 overlapping points) representing 53.5% and 0.1% of each dataset respectively. The added value of these overlaps should not be neglected as they either provide other activity types or help identify certain protein–ligands interactions associated with a lower experimental error.

**Fig. 4:**
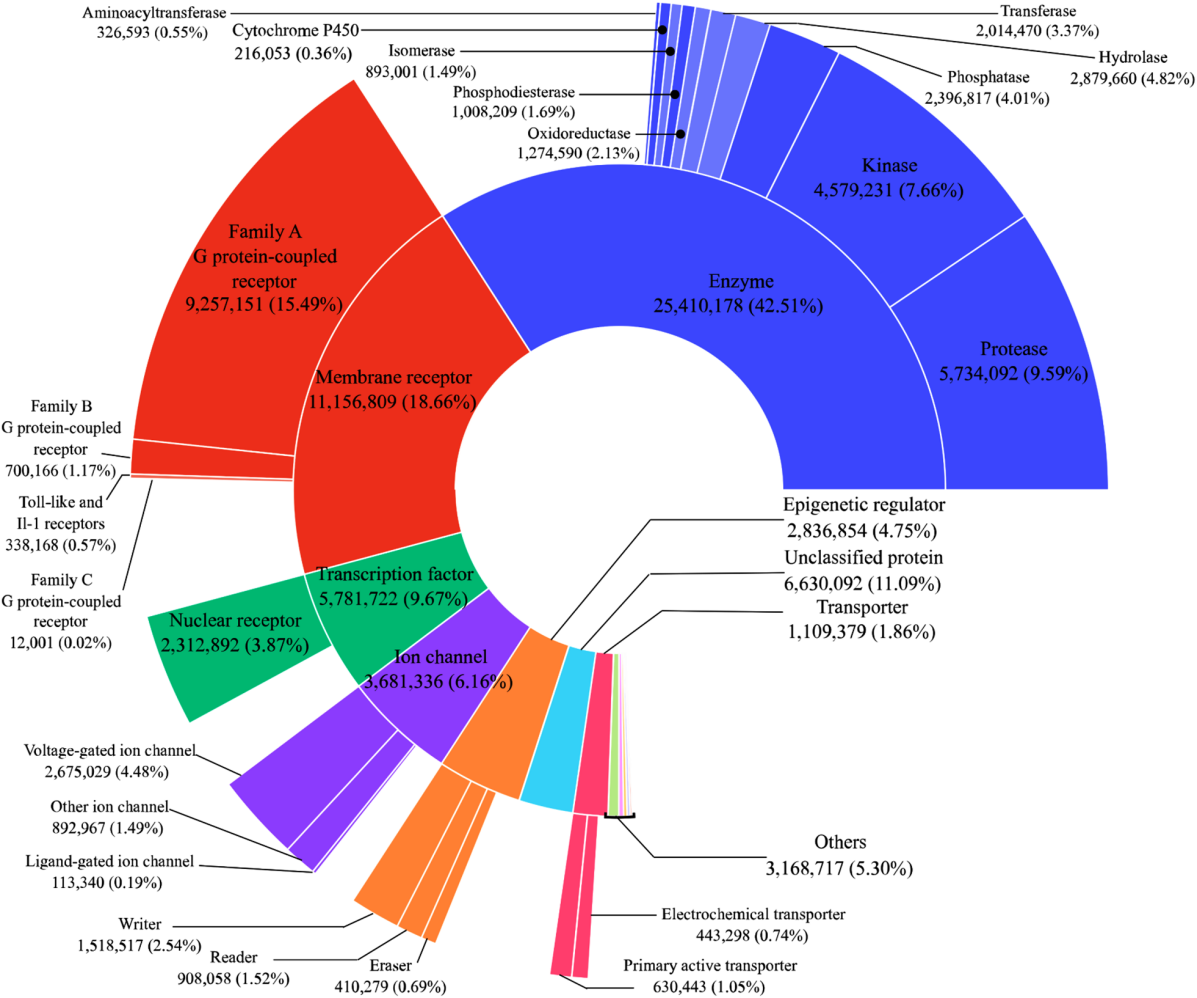
Number of bioactivity data points of protein targets in Papyrus associated with ChEMBL protein classification levels 1 and 2

Concerning protein classification, the two most represented classes are enzymes representing 42.5% of the classified and annotated proteins with more than 25.4 million data points and membrane receptors representing 18.7% with more than 11.1 million entries (Fig. 4; Additional file 3). Family A G protein-coupled receptors represent 15.5% of all data points or 84.0% of those associated with membrane receptors with over 9.2 million data points. Furthermore, proteases represent 9.6% of bioactivity points with more than 5.7 million, and kinases represent 7.7% of the data with more than 4.5 million data points.

### Matching protein data bank data

A total of 9121 unique protein–ligand complexes were matched between the Papyrus dataset and the Protein Data Bank (REST API call made on 2022-07-13; matches available as Additional file 4). These included single structures, but also many examples of multiple protein–ligand complexes. For instance, 28 structures of ZM-241385 bound to the adenosine A_2A_ receptor were retrieved.

### Bioactivity modelling

To exemplify the potential of Papyrus, several data sets were extracted and subjected to QSAR, PCM and DNN modelling (both regression and classification) considering only the high-quality data. A random split and a temporal split scheme were chosen. The latter better assesses the prediction performance of the models [48] and minimizes congeneric series being split between training and test sets. QSAR models were trained on protein targets with sufficient data. This resulted in QSAR models being trained for 12 of the 24 ARs, 9 of the 11 CCRs, 352 of the 476 kinases, 35 of the 37 MRs and 13 of the 33 SLC6. PCM models, able to interpolate between targets, did not require such filtering step and the ensemble of targets was modelled for each subset respectively. As the comparison of the respective performances of molecular descriptors is not the focus of this research, only average metrics are reported in this section.

**Fig. 5:**
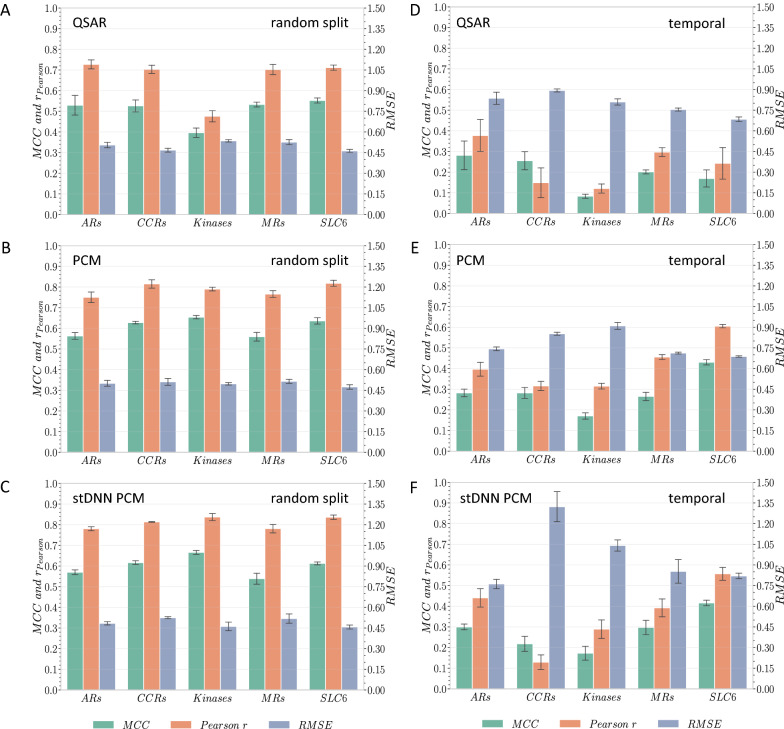
Average performance on the hold-out test set of QSAR, PCM and single-task DNN PCM models using random (**A**–**C** respectively) and temporal splits (**D**–**F** respectively). MCC: Matthews correlation coefficient, RMSE: root-mean-square-error. Error bars indicate standard deviation

The difference in the number of data points in the training sets due to the unequal random and temporal partitions had a very limited effect on the cross-validation performance (Additional file 1: Fig. S3). The major differences in performance between splits were observed in the test sets. The average Matthews correlation coefficient (MCC) of the randomly split QSAR, PCM, and DNN models (Fig. 5A–C) were 0.51, 0.61 and 0.60 respectively. These values corresponded to the observations made by Lenselink et al. [18]. Average Pearson correlation coefficients (Pearson r) increased from 0.66 to 0.79 and 0.81 between QSAR and PCM and DNN models respectively, while root-mean-square error (RMSE) remained constant between 0.73 for QSAR and 0.75 for PCM and DNN models. Concerning the temporal split, average MCC values plummeted to 0.20, 0.29 and 0.30 for QSAR, PCM and DNN models respectively, which is on par with Lenselink et al.’s observations [18]. The average Pearson r decreased to 0.24, 0.42 and 0.36 and RMSE increased to 1.19, 1.17 and 1.44.

These results, being obtained with only data of high quality were on par with previously reported models. Nevertheless, model types and architecture could be optimized to improve performance. Additionally, it is not excluded that further processing and filtering of the Papyrus dataset could improve the overall quality of the obtained models.

### Internal limitations of the dataset

The Papyrus set represents a good benchmark for the community, yet there are some limitations to sets such as Papyrus that we would like to highlight. First, as with most bioactivity data, the set is extremely sparse as only 0.67% of the activity data matrix is available of the total set consisting of 1.25 million compounds and almost 7000 proteins. Data sparsity has been shown to be of importance in the context of selectivity prediction [49] and though several groups have attempted to optimize modelling on these sparse matrices, it remains a challenge. One possible alleviation is the use of active learning to identify information-rich data points that are missing and experimentally determine them [50].

Secondly, as Papyrus is a static dataset, updates or corrections are possible but limited. The authors are planning to update this dataset every year along with the releases of ChEMBL.

Thirdly, stereochemical aspects were discarded in the version of Papyrus this analysis relies on, to ensure that differing molecular standardisation processes of the aggregated sources would not have an impact on the aggregation of activity values. Yet stereochemistry is of the utmost importance, especially when considering activity cliffs [51]. However, cross-set consistency was preferred over potentially erroneous stereochemical data. Nevertheless, a version of the Papyrus dataset in which stereochemistry was conserved is available, though with the footnote that very limited data standardisation was applied and hence usage is generally discouraged compared to the main dataset.

Additionally, the repetition of data in the source datasets was scrutinised and, where possible, only the most recent bioactivity data was kept. For example, the KIBA scores of Tang et al. [12], part of the Merget dataset, were derived from a combination of activity types of ChEMBL version 17 to increase the quality of single measurements and were kept intact in Papyrus. On the contrary, data from ChEMBL version 20 aggregated in the ExCAPE-DB source set, as well as ChEMBL version 21 [25] contained in the Merget et al. [15] source set were disregarded. Hence, though limited, potential duplicates could exist and could bias the aggregated mean and standard deviation for specific compound-target pairs. All in all, these limitations are not unique to Papyrus and apply to any of the secondary sources Papyrus relies on.

Finally, this work overlooked nucleic acids and peptides. For example, around 80 peptide drugs have reached the market and hundreds are in clinical development [52]. Examples include diabetes, cancer, chronic pain, etc. Moreover, peptides have also recently gained interest as a class of antibiotics with a high resistance threshold [53]. Thus, a potential extension of this work could focus on the inclusion of peptides and nucleic acids in the Papyrus dataset.

### Recommendations for use

Based on these observations, the Papyrus++ version of the dataset consisting of measurements with high agreement across multiple assays is recommended to any reader willing to use the data without delving into extensive filtering steps. For those more versed in cheminformatic methods the use of the high-quality full set is recommended depending on the use case scenario.

## Conclusions

We created an openly available large-scale public benchmark set named Papyrus that contains high-quality data aggregated from multiple data sources. This standardised set is primarily used as a reliable data source for modelling ligand–protein interactions. The properties of the set have been investigated and we have demonstrated its usefulness in bioactivity modelling using both QSAR and PCM. It is anticipated that the Papyrus dataset can be exploited in a myriad of ways and filtered or altered for specific research questions. We believe the strength of the dataset lies in its standardisation, normalisation and quality while providing the necessary tools for further manipulation to specific needs.

## Supplementary Information

**Additional file 1.** Additional methods and Figures S1–S3.

**Additional file 2.** Tables S1–S13.

**Additional file 3.** Number of bioactivity data points of protein targets in Papyrus associated with all ChEMBL protein classification levels.

**Additional file 4.** Matches between structures of the Protein Data Bank and Papyrus data.

## Data Availability

The Papyrus dataset, including Papyrus++ and non-standardised stereochemical data can be accessed from https://doi.org/10.5281/zenodo.7019874. The Python scripts, results and figures from which the conclusions herein are derived can be accessed from https://doi.org/10.5281/zenodo.7023464. The custom Python library used to handle the data can be accessed from https://doi.org/10.5281/zenodo.7023086 and is maintained at https://github.com/CDDLeiden/Papyrus-scripts.

## Abbreviations

ARs: Adenosine receptors
CCRs: C–C chemokine receptors
MRs: Monoamine receptors
SLC6: Solute carrier 6 transport family
QSAR: Quantitative structure–activity relationship
PCM: Proteochemometric
DNN: Single-task deep neural networks
InChI: International chemical identifiers
MHFP6: MinHash fingerprint
ECFP6: Morgan fingerprints with radius 3
CDDD: Continuous data-driven descriptors
XGBoost: Extreme gradient boosting
SE_Div_: Sphere exclusion diversity
ChEMBL30: ChEMBL version 30
MCC: Matthews correlation coefficient
Pearson r: Pearson correlation coefficient
