# Supplementary material for: Papyrus: a large-scale curated dataset aimed at bioactivity predictions
Source: J Cheminform. 2023 Jan 6;15:3. doi: 10.1186/s13321-022-00672-x (PMC9824924; doi:10.1186/s13321-022-00672-x)
Supplement: Supplementary file 1 — Additional file 1. Additional methods and Figures S1–S3. [file 13321_2022_672_MOESM1_ESM.docx]

**Supporting information: Papyrus - A large scale curated dataset aimed at bioactivity predictions**

O. J. M. Béquignon^1†^, B. J. Bongers^1†^, W. Jespers^1^, A. P. IJzerman^1^, B. van der Water^1^, G. J. P. van Westen^1*^

1 Division of Drug Discovery and Safety, Leiden Academic Centre for Drug Research, Leiden University, Leiden, The Netherlands

* Corresponding author

Email: [gerard@lacdr.leidenuniv.nl](mailto:gerard@lacdr.leidenuniv.nl) (GJPvW)

† These authors contributed equally to this work

# Additional methods:

## Construction of Papyrus

### ChEMBL

Three levels of quality were defined in the data: high, medium, and low. Data from the different sources were all classified in one of these three classifications. ChEMBL version 30 (ChEMBL30) data^17^ were first split between high- and low-quality data. In total 19,286,751 activity data points measured on 2,157,379 compounds and 14,855 targets were extracted. The following data were deemed as low-quality: data flagged as potential duplicates, of questioned validity (Table S1) - unless errors were confirmed by authors, in which case they were entirely disregarded -, censored, not associated with any pChEMBL value, or of questioned activity (Table S2). Remaining activity data were temporarily regarded as high-quality. The ChEMBL high-quality data originating from PubChem^18^ (source identifier 7) was considered as is but was flagged for later processing of the ExCAPE-DB dataset. Whatever the quality assigned, protein targets were retrieved along with their classifications if they had Uniprot^19^ accessions defined. Accession Q8MMZ4, corresponding to the secondary accession for the Plasmodium falciparum (isolate NF54) cGMP-dependent protein kinase, was manually replaced by its primary accession W7JX98. Using accessions, protein sequences were then obtained from UniProt. Only molecules identified as small molecules and associated with a molecular registry number were kept, then parsed from connection tables and standardised (see Molecular structure standardisation). Activity data of high-quality were reclassified as low-quality if the target type was other than ‘single protein’ or the assay confidence score was 0, 1, 2, 3, 4 or 6 (Table S3). Activity data with assay confidence scores of 5 and 8 were reclassified as medium quality.

If low-quality data were censored, inequality signs of the standard relation were reversed (Table S4) unless expressing an approximation with a tilde, in which case the data were dropped. Standard values of low-quality data with unassigned pChEMBL values were only considered if they had case insensitive standard type of either GI50, Ki, Kd, IC50, EC50 or XC50 and if standard units denoted molar or mass concentrations. Scaling factors were appropriately applied to standard activity values (Table S5), mass concentrations were transformed to molar concentrations and log-scale transformation applied to all concentrations. Only exceptions to data with unassigned pChEMBL values were records with derivatives of the following standard types: pKi, pKd, pIC50, pEC50 or pXC50 (Table S6). For those records, no transformations were applied. Finally, data were flagged on whether activities were derived from IC50, EC50, Ki, Kd or any other data type. The pre-processed ChEMBL30 high-quality data consisted of 1,136,030 activity values, 998,848 compounds and 5,626 targets; the medium-quality data of 99,966 activity values, 263,552 compounds and 2,903 targets; and the low-quality data of 2,139,532 activity values, 516,505 compounds and 5,490 targets.

*ExCAPE-DB*

The ExCAPE-DB dataset consisted of 70,850,163 activity data points of 998,131 compounds measured on 1,667 targets. The set was first discarded of records originating from ChEMBL version 20 or whose assay identifiers were present in the PubChem flagged data of the pre-processed ChEMBL30 mentioned in the previous section. Gene Entrez identifiers were mapped, to unique Swiss-Prot protein sequences using the identifier mapping tool of UniProt^20^. Only four genes were manually mapped: three resolved to multiple reviewed entries and one gene resolved to multiple unreviewed entries (Table S7). ChEMBL30 protein classifications were then assigned to the previously mapped ExCAPE-DB sequences. Deposition dates of the assays were retrieved from PubChem. Data with numeric activity values were considered of high quality and binary data of low quality. Molecular structures failing standardisation (see Molecular structure standardisation) were downloaded from PubChem and standardised afterwards. Finally, low-quality activity data with compound-target pairs present in the high-quality subset were disregarded. The pre-processed ExCAPE-DB high-quality data consisted of 278,226 activity values, 201,644 compounds and 1,535 targets, and low-quality data of 58,445,354 activity values, 650,217 compounds and 646 targets.

*Sharma et al.*

Sharma *et al.*’s dataset^13^, consisting of 258,060 activity data points of 76,017 compounds measured on 8 targets was considered of high quality. Gene names were mapped to unique Swiss-Prot protein sequences using the identifier mapping tool of UniProt and protein classifications retrieved from ChEMBL30. A set of 14 custom reactions (Table S8) were applied to molecular structures failing standardisation (see Molecular structure standardisation), mostly fixing aromaticity-related issues. Years of filing of patents were collected using the Google Cloud BigQuery API on patents public data and manual mapping (Tables S9 and S10) after having fixed erroneous patent numbers (Table S11). Digital object identifiers or PubMed identifiers of source articles were added when missing (Table S12). If activity values were associated with multiple patents, only the first published filed was recorded. Censored activity values or values not associated with case insensitive standard types GI50, Ki, Kd, IC50 or EC50 and their logarithmically-derived counterparts were disregarded. Mass concentrations were transformed to molar concentrations and log-scale transformation applied to all concentrations but those already log-transformed. Finally infinite or activity values lower than 3 and higher than 14 log units were discarded. The pre-processed Sharma data consisted of 73,580 activity values, 40,333 compounds and 8 targets.

*Christmann-Franck et al.*

All the data in the Christmann-Franck *et al.*’s dataset^14^, consisting of 344,788 activity data points containing 2,065 compounds measured on 448 targets, was considered of high-quality. The wrongly assigned Cryptococcus neoformans mitogen-activated protein kinase (CPK1) with accession code P0CP66 was corrected to the Plasmodium falciparum calcium-dependent protein kinase 1 (CDPK1) with accession code P62344. Swiss-Prot sequences were retrieved using accessions and protein classifications retrieved from ChEMBL30. Sequence mutations of the hepatocyte growth factor receptor (MET) and the serine/threonine-protein kinase (B-raf) were corrected to M1250T and V600E respectively and that of the Fibroblast growth factor receptor 1 (FGFR1) was reverted to wildtype. Activity data expressed as proportion of reference activities were discarded. Finally molecular structures were standardised (see Molecular structure standardisation). The pre-processed Christmann-Franck data consisted of 135,948 activity values, 1,669 compounds and 485 targets.

*Klaeger et al.*

Klaeger *et al.*’s dataset^7^, consisting of 5,916 activity data points of 243 compounds measured on 520 targets was considered of high quality. Swiss-Prot sequences were retrieved using HUGO Gene Nomenclature Committee (HGNC) identifiers. If multiple identifiers were assigned the measurement was discarded. Protein classifications were retrieved from ChEMBL30. Apparent Kd values were log-transformed and infinite results disregarded. Finally molecular structures were standardised (see Molecular structure standardisation), with only RDEA-436 failing for its structure was not disclosed. The pre-processed Klaeger data consisted of 5,721 activity values, 228 compounds and 500 targets.

*Merget et al.*

Merget *et al.*’s dataset^15^, consisting of 260,757 activity data points of 47,774 compounds measured on 341 targets was considered of high quality, except for activity values originating from ChEMBL version 22, which were disregarded. Data originating from the Published Kinase Inhibitor Set (PKIS) of GlaxoSmithKline^21^ with activity values of 5 log units were considered as censored and as such reclassified as low-quality data. Unique Swiss-Prot sequences were retrieved using HGNC identifiers^22^, a few of which were manually fixed (Table S13). Protein classifications were retrieved from ChEMBL30. Finally molecular structures were standardised (see Molecular structure standardisation). The Merget pre-processed high-quality data consisted of 127,441 activity values, 1,666 compounds and 239 targets, and low-quality data of 62,642 activity values, 360 compounds and 195 targets.

*Molecular Structure Standardisation*

During the pre-processing of each original dataset parent molecular structures were gathered after a first standardisation using the ChEMBL structure pipeline^23^. Then canonical tautomers were determined using the Pipeline Pilot tautomer enumerator^24^ with tautomerisation of amides enabled. The canonical tautomers were then standardised once again with the ChEMBL structure pipeline after which the parent structures were obtained. Any molecule not parseable from simplified molecular input line entry specification (SMILES) by the RDKit^25^ at any step of the previous workflow was considered failing the standardisation process.

After the individual datasets were processed and aggregated into the Papyrus dataset, molecular structures were standardised once again. This last standardisation ensured normalisation across different sources that each applied different prior standardisation. For instance, tautomerisation tools can alter a compound’s stereochemistry by removing or introducing a chiral centre. To limit the effects of having bioactivity values relating to the same molecular compound having different stereochemistry across sources, a set with removed stereochemistry was created and deemed of higher quality than the set with conserved stereochemistry. Only the higher quality dataset with stereochemistry removed is considered from this point on. Molecular structures, after having removed stereochemistry, were first neutralised with the RDKit by adding or removing hydrogen atoms. Subsequently they were standardised with the ChEMBL structure pipeline after which parent structures were obtained. OpenBabel^26,27^ was then used to recreate dative bonds and to neutralize molecules that were not during the previous step. Tetravalent negatively charged boron atoms were overlooked in the latter stage, making them erroneously pentavalent. This was corrected by detecting these pentavalent boron atoms, removing the newly introduced implicit hydrogen atom and reassigning them a negative formal charge. SMILES of molecules with the same connectivity yet a differing overall charge were InChI-*fied*^28^ with OpenBabel, a step that consists in incorporating the normalisations of the International Chemical Identifier (InChI) canonicalisation process into SMILES. For these molecules this last step removed the dative bonds, that were then recreated using OpenBabel. Then Dimorphite-DL^29^ was used to deprotonate molecules by setting minimum pH to 14.0. This ensured that after the last standardisation step, equivalent to that applied to individual datasets, in which molecules are neutralised, only one charge state of the same molecular species was present in the set.

*Papyrus data aggregation*

The processed ChEMBL30 high-, medium- and low-quality, ExCAPE-DB high- and low-quality, Sharma, Christmann-Franck, Klaeger and Merget datasets were aggregated together. The first step consisted in ensuring that the activity of any compound-target pair was contained within 3 to 14 log units. Then compound-target pairs were uniquely identified by a concatenation of the compound’s connectivity and of the target accession along with its mutations if any. All activities relating to the same compound-target pair were then filtered depending on the highest data quality available for that pair. For instance, if high-quality activities were identified, any data point deemed of medium to low quality was filtered out. Considering censored activity values, the data was filtered out if contradictory relations were identified, if multiple censored thresholds were given the highest was retained for lower bounds, and lowest for higher bounds. During this filtering step, all patents and journal articles associated with the activity of a compound-target pair were gathered whatever the quality and only the first published or filed was retained. Finally, activity values were aggregated and mean averages, medians, standard errors of the mean, standard deviations and mean average distances were calculated for each unique compound-target pair.

# Additional Figures:


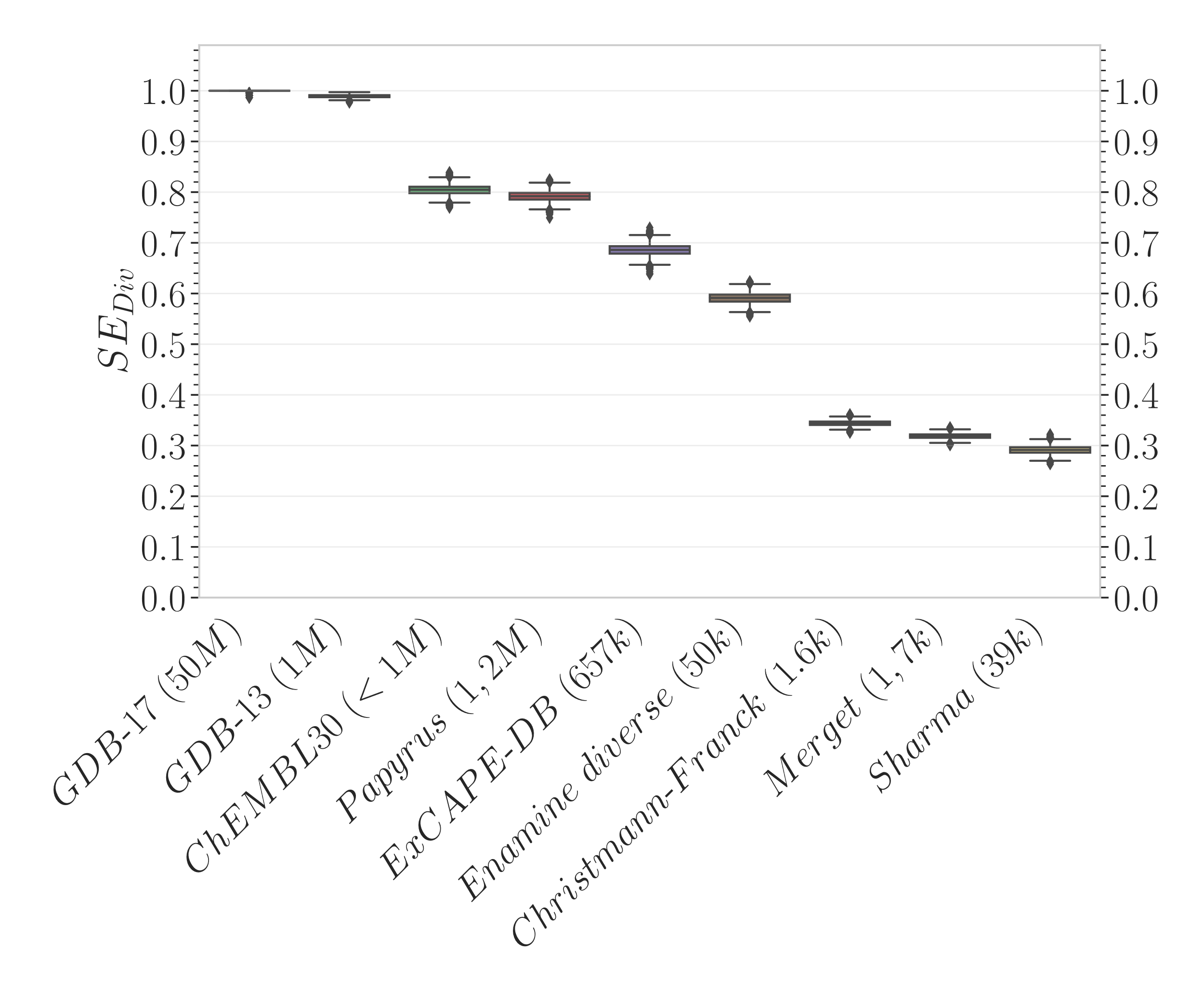


Additional figure 1: Sphere exclusion diversity (SE_Div_) of randomly sampled subsets of 1,500 molecules of the Papyrus dataset, its source subsets and reference virtual libraries GDB17, GDB-13 and Enamine synthetically accessible diversity set. The Klaeger dataset was excluded from the visualisation as it contains only 228 molecules.


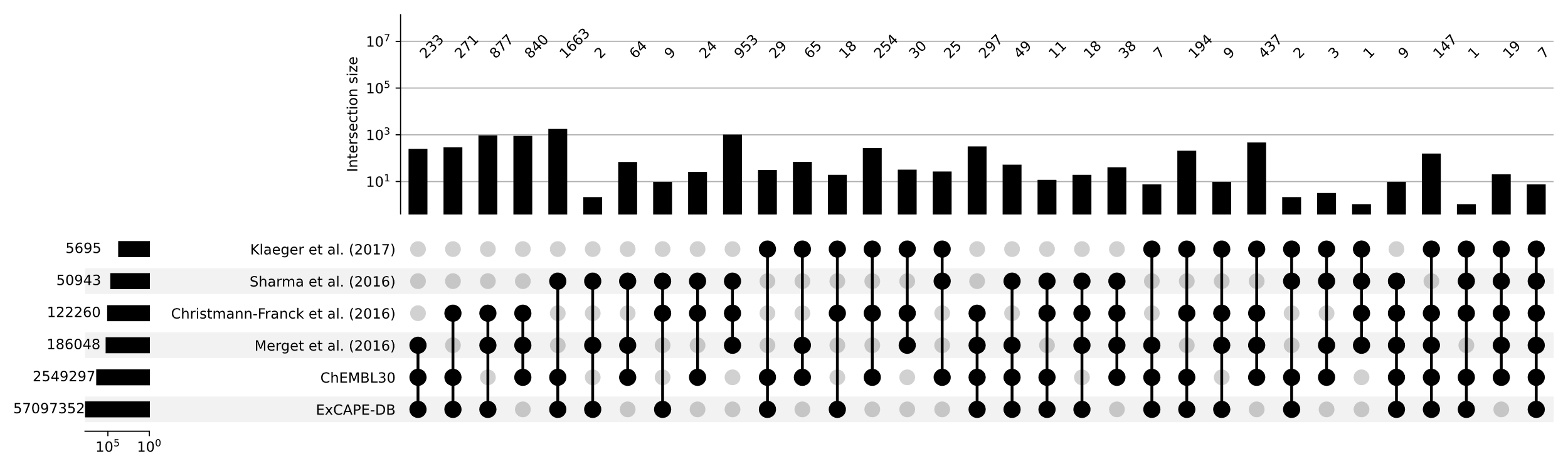

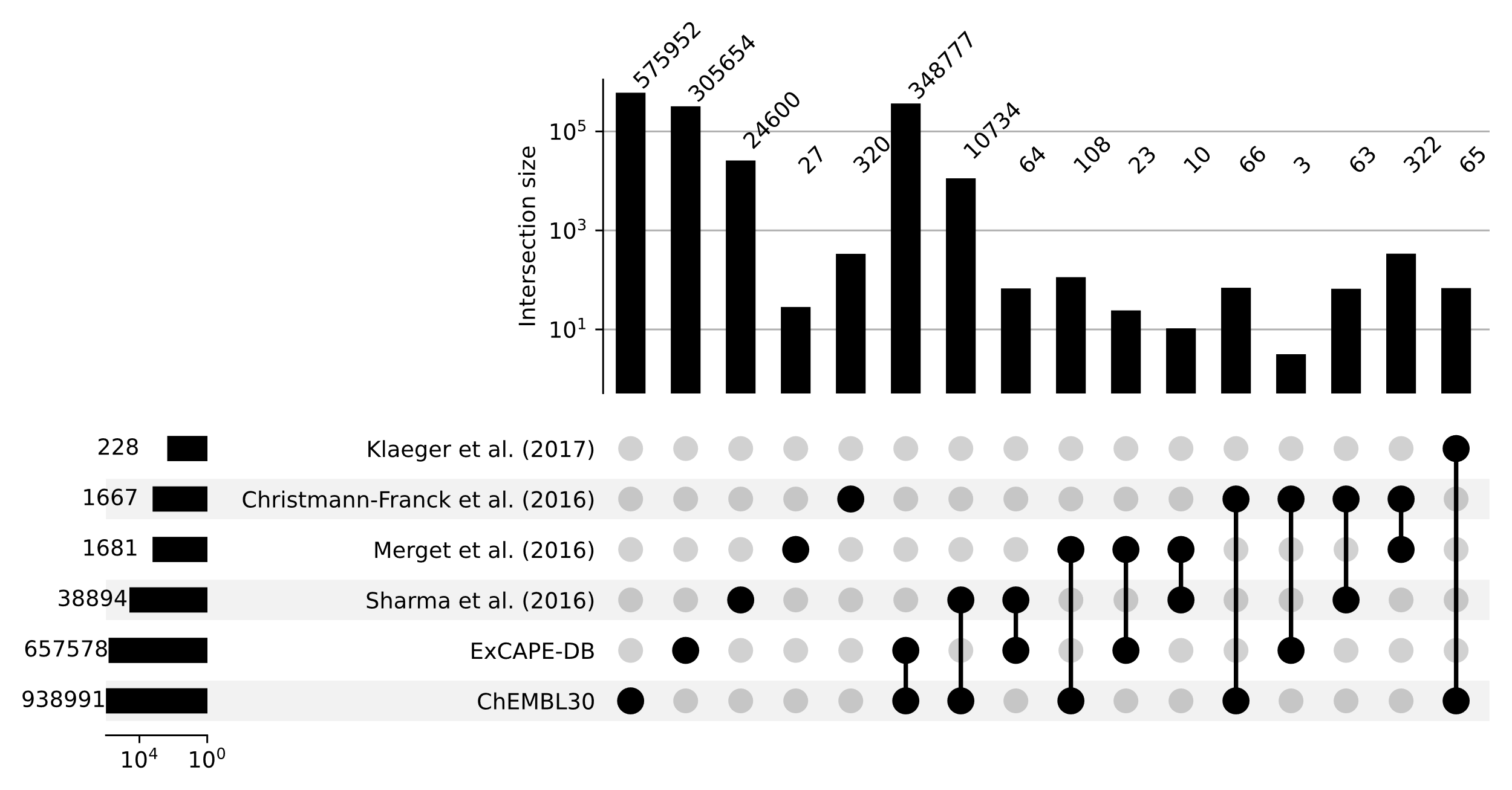

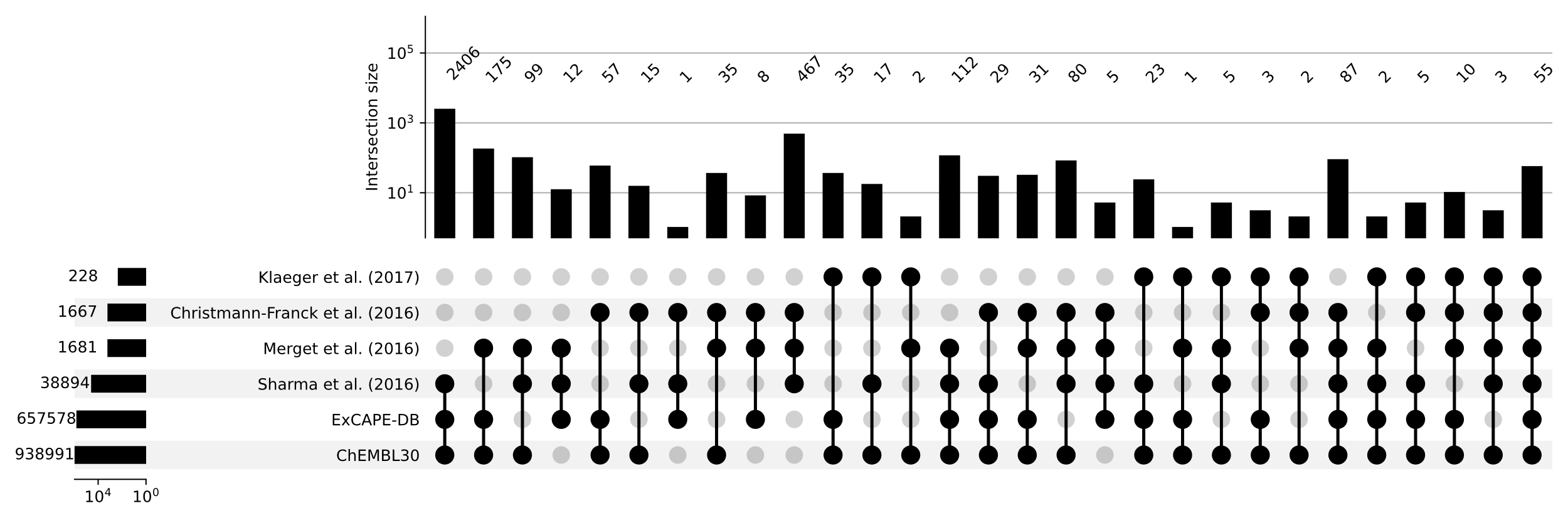

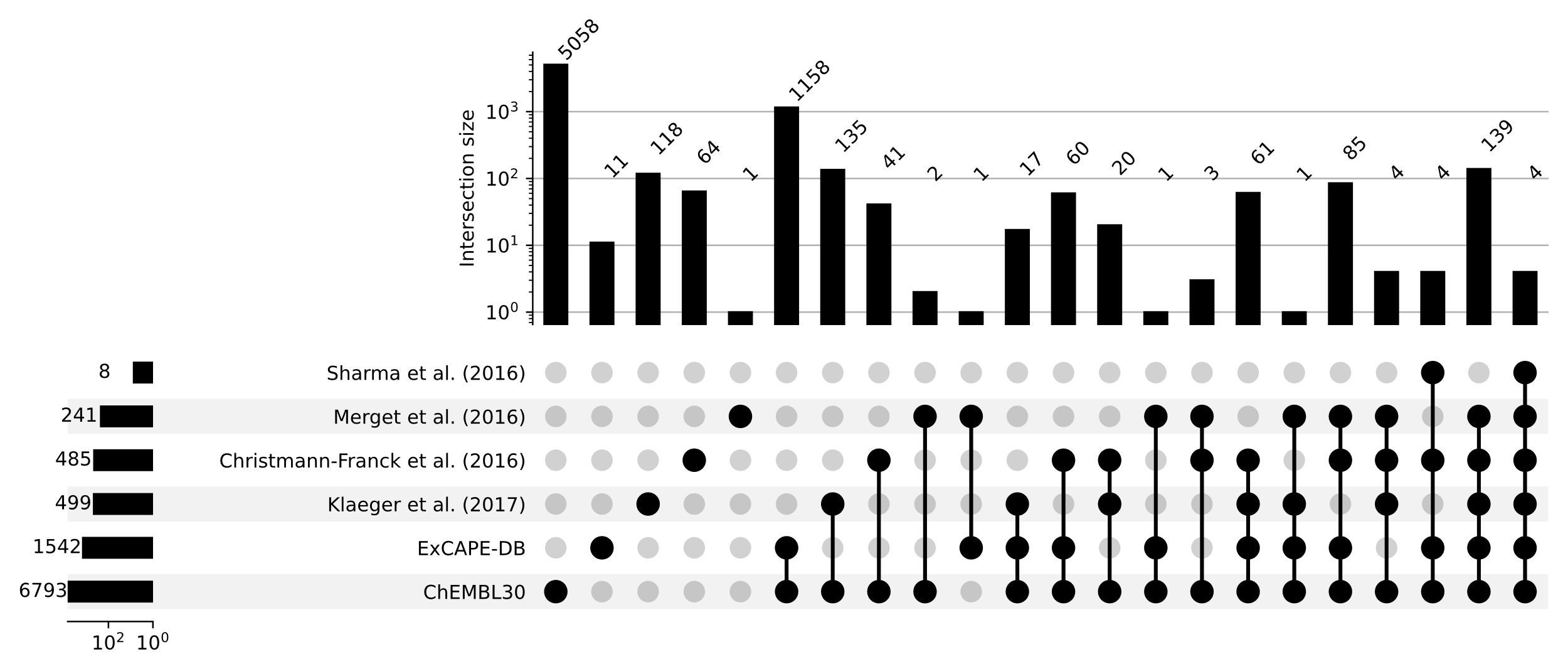


A

B

C

D

Additional figure 2: Overlaps of the activity datapoints between more than two of the aggregated datasets (A), of compounds between up to two (B) and more than two datasets (C) and of protein targets (D). Shown on the left side are the number of points in each dataset, with the number on the top shows how many entries are found in that particular dataset or overlap of datasets.


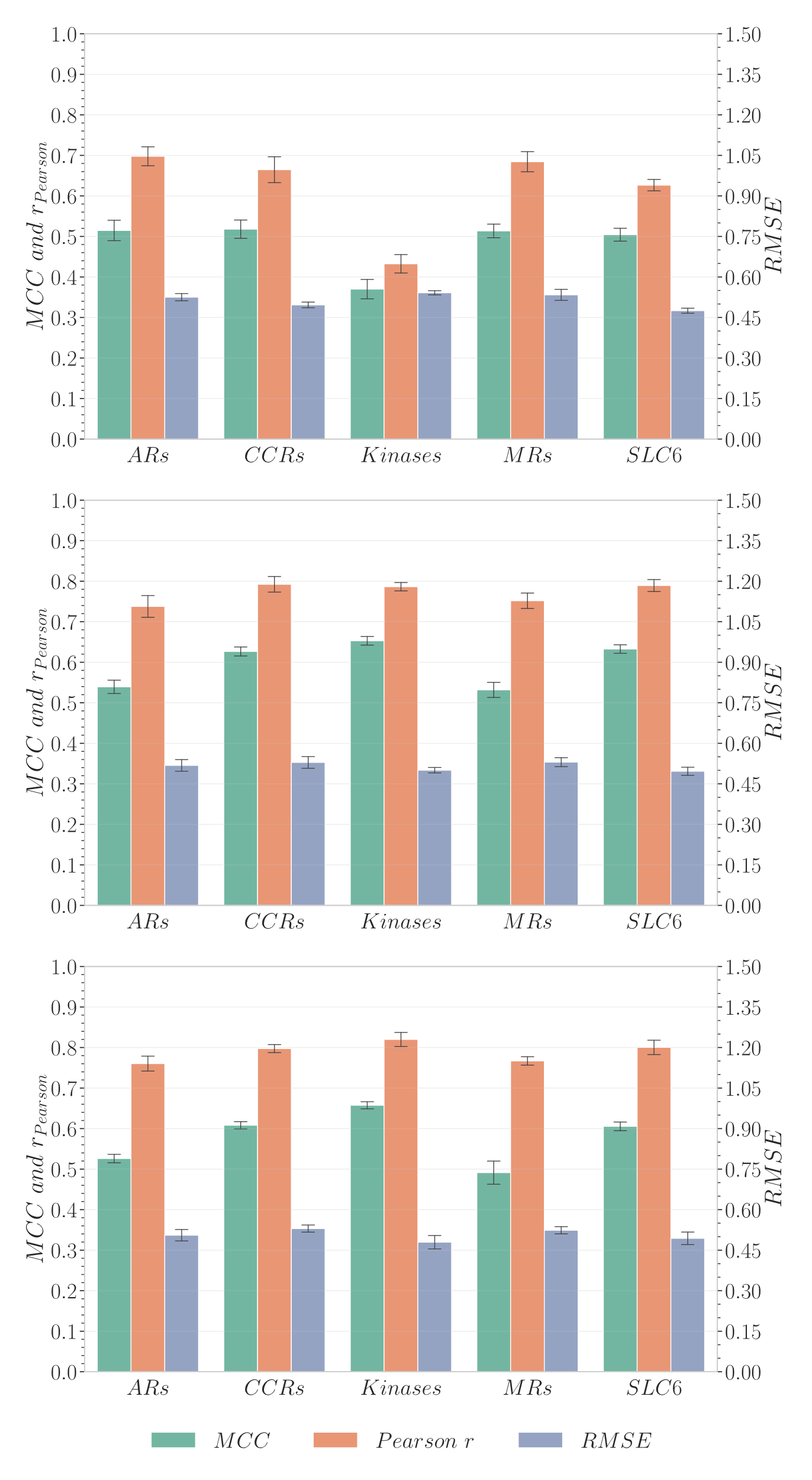


A

B

C

QSAR

random split

PCM

random split

stDNN PCM

random split


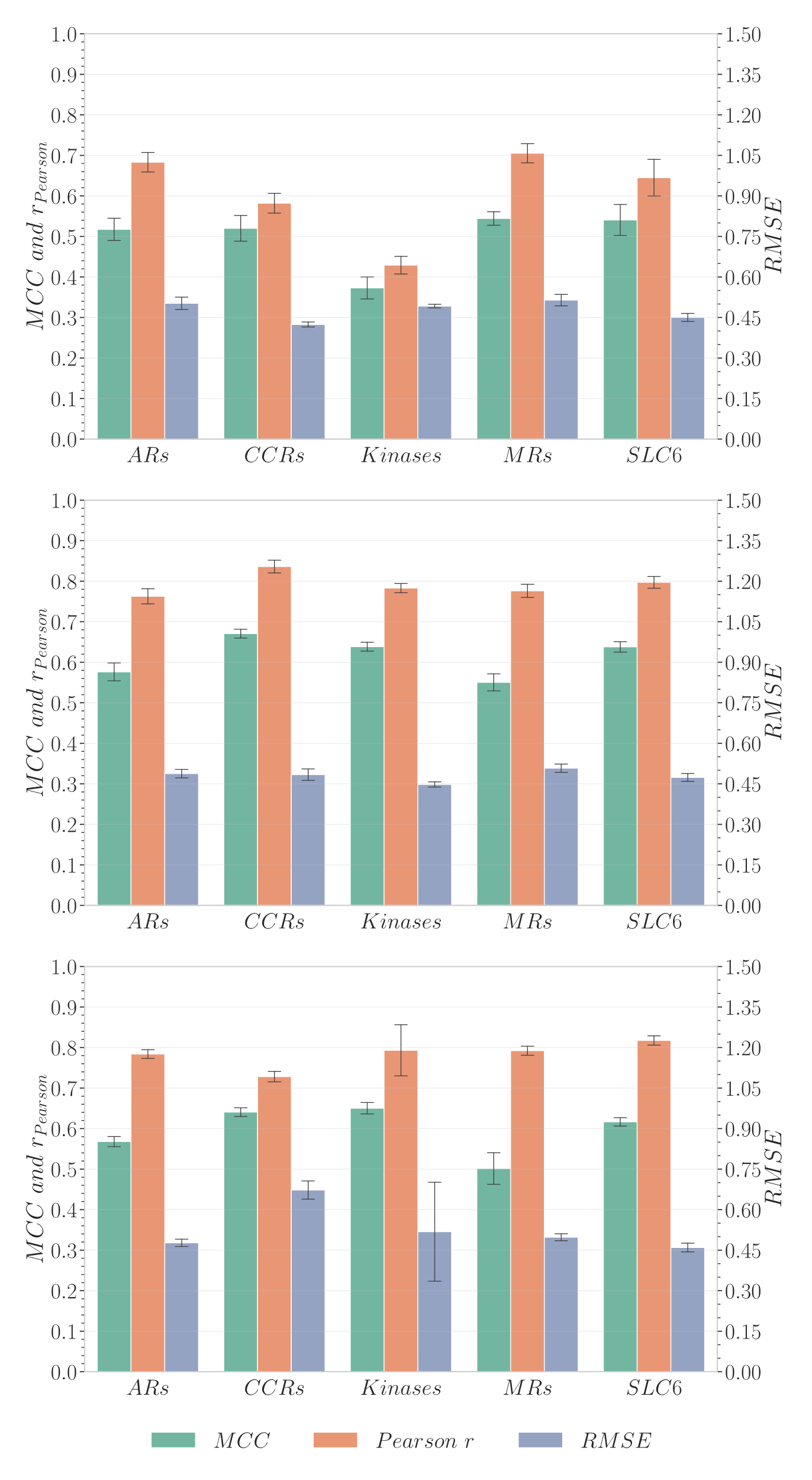


D

E

F

QSAR

temporal split

PCM

temporal split

stDNN PCM

temporal split

Additional figure 4: Average cross-validation performance on training set of QSAR, PCM and single-task DNN PCM models using random (A, B and C respectively) and temporal splits (D, E and F respectively). MCC: Matthews correlation coefficient, RMSE: root-mean-square-error. Error bars indicate standard deviation.
